# Role of epithelial mesenchymal transition (EMT) in chronic obstructive pulmonary disease (COPD)

**DOI:** 10.1186/1465-9921-14-120

**Published:** 2013-11-06

**Authors:** Sukhwinder Singh Sohal, Eugene Haydn Walters

**Affiliations:** 1 NHMRC Centre of Research Excellence for Chronic Respiratory Disease, School of Medicine, University of Tasmania, Hobart 7000, Australia; 2 NHMRC Centre for Research Excellence in Chronic Respiratory Disease, School of Medicine, University of Tasmania, MS1, 17 Liverpool Street, Private Bag 23, Hobart, Tasmania 7000, Australia

**Keywords:** Epithelial mesenchymal transition, Small airways, Large airways, COPD, Lung cancer, Vimentin

## Abstract

Small airway fibrosis is the main contributor to physiological airway dysfunction in COPD. One potential mechanism contributing to small airway fibrosis is epithelial mesenchymal transition (EMT). When associated with angiogenesis (so called EMT-Type-3) it may well also be the link with the development of cancer, which is closely associated with COPD and predominantly in large airways. In a recent study published in Respiratory Research, Qin Wang and colleagues investigated the role of urokinase plasminogen activator receptor (uPAR) in EMT in small airway epithelium of COPD patients. However, there are some issues with the paper which we wish to comment on.

## Letter to the Editor

### Dear Editor

We read with interest the recent paper by Qin Wang and colleagues published in Respiratory Research (2013, 14:67, doi:10.1186/1465-9921-14-67), addressing epithelial mesenchymal transition (EMT) in COPD airways [1]. This is an important new area and it is quite reassuring that leading respiratory journals are now recognising the potential importance of EMT in pathogenesis of chronic obstructive pulmonary disease (COPD) and its deadly consequences. In this paper the authors conclude that increased urokinase plasminogen activator receptor (uPAR) expression in the small airway epithelium of patients with COPD is a manifestation of an active EMT process [1]. However, we have some issues with the paper.

Firstly, in the discussion Qin Wang and et al. questioned the relevance of our findings on active EMT in large airways of COPD patients. We agree that EMT-type-2 may be involved in pathogenesis of small airway and obliteration. However, a second major feature of COPD, namely, the striking vulnerability of early stage COPD to development of lung cancer [2-4], is also poorly understood. We have suggested that EMT-type-3 in larger airways provides an explanation [2-9]. Wang and et al. have missed this point in our paper, by being overly focused on EMT in small airways, EMT also seems to feature in idiopathic pulmonary fibrosis (IPF) and bronchiolitis obliterans syndrome (BOS) [2-11].

**Figure 1:**
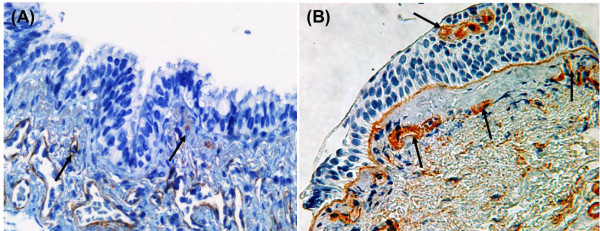
**Airway tissue stained for type-IV collagen to highlight blood vessels. (A)** Small airway section, black arrows indicating vessels in the lamina propria with no vessels in the Rbm or epithelium, typical of EMT Type-2; **(B)** bronchial biopsy section from a matched large airway, with black arrows indicating vessels with in Rbm and also penetrating into the epithelium, typical of EMT Type-3.

EMT when associated with increased angiogenesis of the reticular basement membrane (Rbm) and indeed epithelium itself (Figure 1), leads to the formation of a pro-cancer stroma (EMT-type-3) in contrast to mainly fibrosis-associated EMT-type-2 which lacks angiogenesis [2-4,7-9,12]. It is thought that this aberrant vessel plexus gives immune protection to developing malignant cells by preventing egress and local activity of natural killer (NK) cells [13,14]. Fibroblasts originated from type-3 EMT are designated as “cancer-associated fibroblasts” [15]. This is well described in other epithelial malignancies [16]. It is of interest and relevance in this context that over 90% of human cancer arises in epithelia (e.g. breast, colon, stomach, liver, prostate, ovary/fallopian tube, bladder etc.), and the involvement of EMT in all of these may be a central paradigm [16]. It is pertinent that up to 70% of lung cancer occurs in the context of mild-to-moderate COPD [17-19], and COPD-related cancer may well be just another example of this core principle of unstable epithelium in the context of tissue inflammation and/or chronic stimulation.

Our second major issue is the quality of immunostaining of small airway sections for EMT bio-markers in the Wang paper. In Figure 1A, the serial sections from COPD patients stained for E-cadherin (b) and vimentin (d) are not convincing. There is indeed decreased expression of E-cadherin in the epithelium but the authors failed to point out what other cells are staining for E-cadherin nearly 150 microns deep in the lamina propria (LP). If they have migrated from the epithelium they should have lost E-cadherin and should be positive for vimentin in the LP. This suggests some artefact; perhaps the authors should have considered double immunostaining for these markers. In addition, the non-smoker small airway tissue section demonstrates a quite hyperplastic appearance of the epithelium, suggesting aberrant phenotyping.

Finally, the authors show correlations between the expression of cell mesenchymal markers in small airways and lung function for all the 78 subjects together. However, this includes quite distinct groups, including normal, which are bound to give such a relationship. What is needed is an analysis of obstructed participants only, to see if greater expression of markers is related to increased loss of function. This seems unlikely from the data shown.

Inspite of our reservations, this is still an important study which highlights the potential of EMT to be central to COPD pathophysiology. Acceptance of this new concept will change the very way we think about this disease process and its nasty clinical consequences.

## Abbreviations

BOS: Bronchiolitis obliterans syndrome; COPD: Chronic obstructive pulmonary disease; EMT: Epithelial mesenchymal transition; IPF: Idiopathic pulmonary fibrosis; LP: Lamina propria; NK: Natural killer cells; Rbm: Reticular basement membrane; uPAR: Urokinase plasminogen activator receptor

## Competing interests

Authors declare that they do not have any competing interests.

## Authors’ contributions

SSS: literature search, figures, performed the histological analyses, data collection, data interpretation and writing. EHW: design of study, clinical assessments, overview of all analyses, data interpretation and writing. Both authors read and approved the final manuscript.
